# Impact of Aeolian Dry Deposition of Reactive Iron Minerals on Sulfur Cycling in Sediments of the Gulf of Aqaba

**DOI:** 10.3389/fmicb.2017.01131

**Published:** 2017-06-20

**Authors:** Barak Blonder, Valeria Boyko, Alexandra V. Turchyn, Gilad Antler, Uriel Sinichkin, Nadav Knossow, Rotem Klein, Alexey Kamyshny

**Affiliations:** ^1^Department of Geological and Environmental Sciences, Faculty of Natural Sciences, Ben-Gurion University of the NegevBeer Sheva, Israel; ^2^Department of Earth Sciences, University of CambridgeCambridge, United Kingdom

**Keywords:** Red Sea, Gulf of Aqaba, aeolian dust deposition, highly reactive iron, manganese, sulfide oxidation intermediates, cryptic sulfur cycle

## Abstract

The Gulf of Aqaba is an oligotrophic marine system with oxygen-rich water column and organic carbon-poor sediments (≤0.6% at sites that are not influenced by anthropogenic impact). Aeolian dust deposition from the Arabian, Sinai, and Sahara Deserts is an important source of sediment, especially at the deep-water sites of the Gulf, which are less affected by sediment transport from the Arava Desert during seasonal flash floods. Microbial sulfate reduction in sediments is inferred from the presence of pyrite (although at relatively low concentrations), the presence of sulfide oxidation intermediates, and by the sulfur isotopic composition of sulfate and solid-phase sulfides. Saharan dust is characterized by high amounts of iron minerals such as hematite and goethite. We demonstrated, that the resulting high sedimentary content of reactive iron(III) (hydr)oxides, originating from this aeolian dry deposition of desert dust, leads to fast re-oxidation of hydrogen sulfide produced during microbial sulfate reduction and limits preservation of reduced sulfur in the form of pyrite. We conclude that at these sites the sedimentary sulfur cycle may be defined as cryptic.

## Introduction

Reduction–oxidation (redox) reactions in marine sediments are the only source of metabolic energy for microorganisms in the absence of light. The dominant electron donor in marine sediments is organic carbon due to its high metabolic energy yield and general availability in marine environments (Boudreau, 1992; Orcutt et al., 2011). Microbially mediated oxidation of this organic carbon utilizes electron acceptors according to the metabolic energy yield of the respective redox reaction. Electron acceptors are generally consumed according to the Gibbs free energy yield of respiratory pathways associated with organic matter oxidation (Froelich et al., 1979). Depending on the availability and lability of organic carbon in the sediment, and the concentration and speciation of electron acceptors, zones characterized by the reduction of various electron acceptors may overlap (Canfield et al., 1993; Thamdrup, 2000; Hansel et al., 2015). In marine and marginal marine sediments, the oxidation of organic carbon coupled to iron reduction and microbial sulfate reduction are of key importance. Recent work has shown that the sedimentary iron and sulfur cycles may be coupled in interesting and complicated ways (Holmkvist et al., 2011; Mills et al., 2016).

There are three possible fates for hydrogen sulfide produced during microbial sulfate reduction. The first fate is removal through precipitation as iron monosulfide followed by conversion to pyrite (Burdige and Nealson, 1986). The second fate is possible in iron-limited environments: hydrogen sulfide can be incorporated into sedimentary organic matter through polysulfide formation (Werne et al., 2008; Amrani, 2014). The third fate is re-oxidation by chemical or microbially mediated processes to sulfide oxidation intermediates (S_n_^2-^, S^0^, S_2_O_3_^2-^, SO_3_^2-^, and S_4_O_6_^2-^) and/or to the terminal oxidation product, sulfate (Chen and Morris, 1972; Yao and Millero, 1996; Lichtschlag et al., 2013; Holmkvist et al., 2014). Thus, if microbial sulfate reduction takes place in sediments rich in electron acceptors such as reactive Fe(III) (hydr)oxides and manganese oxides (MnO_2_), hydrogen sulfide may be either totally reoxidized to sulfate or only partially preserved as trace amounts of pyrite in the sediment. If re-oxidation of hydrogen sulfide is fast enough, aqueous sulfide concentrations may remain low and no products of microbial sulfate reduction (e.g., iron sulfide, pyrite, organic sulfur) are preserved. In this case the sulfur cycle has been called “cryptic” (Turchyn et al., 2006; Holmkvist et al., 2011; Hansel et al., 2015; Mills et al., 2016).

Transformation of sulfate to hydrogen sulfide is associated with a large sulfur isotopic fractionation (Harrison and Thode, 1958); up to ε≈70‰ at ambient temperature has been suggested using models of microbial metabolism (Farquhar et al., 2003; Brunner and Bernasconi, 2005), sulfur isotopic fractionation up to ε = 66‰ has been measured in microbial pure cultures (Sim et al., 2011a), and sulfur isotope fractionation up to 77‰ has been measured in deep ocean sediments at elevated temperatures (Rudnicki et al., 2001). The extent of sulfur isotope fractionation depends on sulfate concentrations, microbial sulfate reduction rate, substrate type, and temperature (Habicht et al., 2002; Sim et al., 2011b). In the case of a cryptic sulfur cycle, where most hydrogen sulfide is re-oxidized to sulfate and neither free hydrogen sulfide nor pyrite are present, sulfur isotopes are less useful for study of the biogeochemical sulfur cycle because they are quantitatively conserved and do not necessarily exhibit a large sulfur isotope fractionation (Mills et al., 2016). In this case study, the oxygen isotope composition of sulfate has been shown to be a very powerful tool for studying the redox cycling of sulfur. Similar to sulfur isotopes, oxygen isotopes in dissolved sulfate (δ^18^O_SO4_) also increase during microbial sulfate reduction as the light ^16^O isotope is preferentially reduced, but at some stage stop increasing and reach a value that is in oxygen isotopic equilibrium with water (Fritz et al., 1989; Böttcher et al., 1999). As sulfate and water do not exchange oxygen isotopes easily, this observed oxygen isotope exchange is attributed to the intracellular exchange of oxygen atoms between sulfur intermediate species (such as sulfite) and water (Mizutani and Rafter, 1973; Fritz et al., 1989). The observed ^18^O isotope enrichment over the isotopic composition of water (magnitude 22 - 30‰) reflects intracellular oxygen isotope exchange (Böttcher et al., 1998, 1999; Brunner et al., 2005, 2012; Turchyn et al., 2006, 2010; Antler et al., 2013). Therefore, if a significant fraction of sulfate is reduced and reoxidized to sulfate, exchange of oxygen isotopes between sulfate and water can be detected, and thus the oxygen isotopic composition of sulfate may be a proxy for any quantitative cycling of sulfur such as during a cryptic sulfur cycle (Bishop et al., 2013; Johnston et al., 2014).

Airborn dust is an important source of nutrients and essential metals to wide areas of the ocean (Duce et al., 1991). The input of mineral dust may affect seawater biogeochemistry and sedimentation processes (Jickells, 1995; Chen et al., 2008). Aeolian dust deposition is especially significant in marine systems that are situated in arid environments where fluvial input is limited. Wind-transported dust with high iron and manganese content, which mainly consists of minerals derived from arid and semi-arid mid-latitude regions in the Northern Hemisphere, has been suggested as a crucial factor that may control primary productivity in high nutrients - low chlorophyll marine ecosystems (Duce and Tindale, 1991; Street and Paytan, 2005). After deposition of dust at the ocean surface, only a small fraction of the aeolian iron flux becomes bioavailable due to the low solubility of the colloidal and particulate Fe(III) (mostly between 1 and 12%) that comprises the bulk of the aerosol iron pool (Mahowald et al., 2005). In the Red Sea, ≤2% of dry-deposited aerosol iron is dissolved during the transport of dust particles to the sediment (Chen et al., 2006).

High fluxes of aeolian dry deposition of dust from adjacent deserts makes the Gulf of Aqaba an ideal playground for the study of the impact of high concentrations of reactive iron and manganese on sulfur cycling in sediments in an oligotrophic marine system. In this work, we report on the concentrations of iron, manganese and sulfur (in various valence states) as well as the isotopic composition of sulfur in the sediments, across a range of water depths, including a site influenced by anthropogenic activity. We evaluate the importance of the reoxidative part of the sulfur cycle, and how this varies with changes in water depth, and identify a cryptic sulfur cycle at the deep-water sites.

## Study Site

**FIGURE 1:**
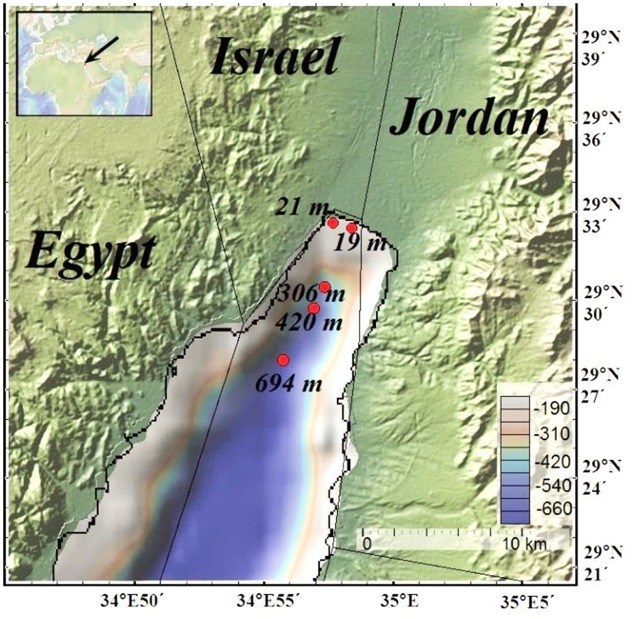
Map of the Gulf of Aqaba. Red dots represent sampling sites (map was generated using GeoMapApp, http://www.geomapapp.org).

The Gulf of Aqaba is 180 km long, 6 to 25 km wide and up to 1850 m deep (Ben-Avraham et al., 1979) (**Figure 1**). A transform fault located at the center of the Gulf of Aqaba causes steep-slope topography. The limited water influx from the Red Sea through the narrow (800 m wide) and shallow (80–250 m depth) Straits of Tiran results in high water column temperature (21–27°C) throughout the year, as warm surface waters flow from the Red Sea into the Gulf and deep waters flow out from the Gulf into the Red Sea (Ben-Avraham et al., 1979; Biton and Gildor, 2011). During the summer months, the water column is stratified and water mixing occurs only to a depth of 20 m. During winter, the water column typically mixes to a depth of 300-400 m but can be mixed down to 860 m depth during exceptionally cold winters (Genin et al., 1995). Nevertheless, the bottom waters of the Gulf of Aqaba are oxic throughout the year with a reported oxygen minimum of 168 μM at 800 m (Reiss and Hottinger, 1984; Badran, 2001). The Gulf of Aqaba is an oligotrophic marine system. Primary production in photic zone (upper 170 m) of the Gulf of Aqaba varies in the range of 0.05–3.38 mg C m^-3^ h^-1^ (Levanon-Spanier et al., 1979; Stambler, 2006). The productivity rates are limited due to the seasonal nutrient (N, P) depletion during summer stratification (Reiss and Hottinger, 1984; Li et al., 1998; Labiosa et al., 2003).

Particulate input is mostly of aeolian origin and other inputs are relatively low due to scarse local rainfall (22 mm y^-1^). Recently, it was reported that possible sources of fluvial material to the Gulf are desert flash floods from the Arava desert after rare rainfall events (Katz et al., 2015), but deposition of this fluvial material would impact only sediments in the immediate vicinity of the shoreline. However, in the vicinity of coral reefs the coarse sediments are nearly all biogenic, as depositional rates of terrigenous material from wadies are equal to coral growth (Reiss and Hottinger, 1984). Reported iron content in suspended flashflood sediments is 41 mg g^-1^ with total inflow into the Gulf of 10 g Fe m^-2^ (179 mmol m^-2^) (Katz et al., 2015). Sediments at the north shore of the Gulf, which is adjacent to Arava Valley, are fluvial silts and fine sands with a mean grain size of 257 μm and c.a. 20–23% silt (Katz et al., 2002). Sediments overlain by deep waters (825 m) are characterized by smaller grain size, with a mean grain size of 45 μm and total organic carbon of 0.4% (Rasheed et al., 2003).

Sedimentation rates in the deep waters (up to 826 m) near the Jordanian coast are 42–72 cm ky^-1^ (Al-Rousan et al., 2004). Lower rates, 4–10 cm ky^-1^ were reporetd by Reiss et al. (1980) at <900 m depth. Much higher rates, 220–330 cm ky^-1^, were reported for shallow (10 m water depth, coral reef) sites by Larsen (1978). The strong variation in sedimentation rates in the Gulf of Aqaba may be caused by different degrees of resuspension, which depends on the textural properties of the sediments (e.g., finer sediments with low density are resuspended to larger areas) as well as on storm events (Schumacher et al., 1995; Al-Rousan, 1998).

The Gulf of Aqaba is strongly affected by dust storm events, which supply dust particles with iron, aluminum and manganese from adjacent (e.g., the Negev Desert, the Sinai, and Arabian Peninsulas) and distant (e.g., the Sahara) arid environments (Chase et al., 2006). The total deposition rate of aeolian mineral dust to the Gulf of Aqaba is 34.7 g m^-2^ year^-1^ (Al-Taani et al., 2015), whereas the aerosol iron deposition flux varies in range of 1.5–116 μmol m^-2^ d^-1^ with a mean of 10 μmol m^-2^ d^-1^ (Chase et al., 2006). Inputs of terrigenous sedimentary material are restricted to seasonal hyperpycnal flows, which may reach reef sediments during rare rain events.

Along the northern shore of the Gulf of Aqaba near the Israel-Jordan border, a fish farm (‘Ardag’) operated from 1989 to 2008. The fish farm contributed large amounts of organic carbon to the previously organic matter-poor sediments. In 2002, total organic carbon content as high as 6.85% was detected in the sediments at the fish farm site (Black et al., 2012).

## Materials and Methods

### Sampling

Five sampling campaigns were carried out between May 2012 and February 2013 at water depths of 19 m (site RS-II-19, ex-fish farm location), 21 m (site RS-V-21), 306 m (site RS-IV-306), 420 m (site RS-III-420), and 694 m (site RS-I-694) (**Figure 1** and **Table 1**). Sampling sites map was generated using GeoMapApp^1^ (Ryan et al., 2009). At the deep-water sites, cores were retrieved by a multicorer from the IUI-Eilat research vessel. Near-shore sampling was performed manually by divers. The core liners were 50 cm long Perspex tubes with 9.5 cm internal diameter. Three cores were retrieved during each sampling. These cores were used for high resolution dissolved oxygen concentration measurements (Core 1), total sediment composition analysis (Core 2), pore-water extraction and chemical analyses (Core 3).

**Table 1:** Water depths and coordinates of sampling sites.

Sampling site	Water depth, *m*	Coordinates
RS-II-19	19	29°32′30.6″N/34°58′24″E
RS-V-21	21	29°32′41.4″N/34°57′45.6″E
RS-IV-306	306	29°30′58.2′N/34°57′30.6″E
RS-III-420	420	29°30′12.6′N/34°56′58.2′E
RS-I-694	694	29°28′3″N/34°55′41.4″E


### Pore-Water Extraction

The pore-waters were extracted at 4 cm intervals by Rhizon samplers (MacroRhizon, 9 cm long, 4.5 mm diameter, Rhizosphere Research Products, Netherlands) with a filter pore size of 150 ± 30 nm, under a nitrogen atmosphere in a glove box (Seeberg-Elverfeldt et al., 2005).

The first 1 mL of every sample was discarded to avoid sample contamination. The volume of pore-water extracted at each core depth was c.a. 50–80 ml. The extraction of large pore-water volumes was crucial in order to perform numerous geochemical analyses for a variety of dissolved species. The vertical overlap between pore fluid samples retrieved with the Rhizons was calculated according to Seeberg-Elverfeldt et al. (2005) and was found to be in the range of 0–14%.

### Pore-Water Analysis

Ferrous (Fe^2+^) iron was quantified spectrophotometrically by the ferrozine method according to Stookey (1970). The minimum detection limit (MDL) of this method is 1 μmol L^-1^. Manganese in pore-waters was also measured spectrophotometerically (LaMotte, SMART Spectro) using the 1-(2-Pyridylazo)-2-naphthol (PAN) method (LaMotte test kit 3660-sc) according to Goto et al. (1977). Ascorbic acid was used to reduce total manganese to dissolved Mn^2+^.

Hydrogen sulfide was measured by two methods. For samples with hydrogen sulfide concentration >1.5 μmol L^-1^ samples were pre-treated with 50 g L^-1^ zinc acetate at a ratio of at least 3:20 (V/V) immediately after sampling and the concentration was quantified by the methylene blue method according to Cline (1969). This method accounts for the following species: H_2_S, HS^-^, S^2-^, S(II) in polysulfides, as well as nanoparticles of acid-soluble metal sulfides (e.g., MnS, FeS), which can pass through the pores of the Rhizon sampler. The MDL was 1.5 μmol L^-1^. For samples with hydrogen sulfide concentration <1.5 μmol L^-1^, quantification was performed by high-precision liquid chromatography (HPLC, Agilent Technologies 1260 Infinity) with fluorescence detection after derivatization with monobromobimane (Kosower et al., 1979; Fahey and Newton, 1987; Zopfi et al., 2004). The sulfur species measured by this method are: H_2_S, HS^-^, S^2-^ and, possibly, the S(II) of polysulfides. The MDL for the sum of these species was 2 nmol L^-1^. Dissolved zero-valent sulfur (ZVS, includes dissolved and colloidal elemental sulfur and well as polysulfide zero-valent sulfur) was quanified by cyanolysis according to Kamyshny (2009a). The MDL was 60 nmol L^-1^. The concentrations of polysulfides were below the detection limit of chromatographic quantification after derivatization to dimethylpolysulfanes with methyl trifluoromethanesulfonate (MDL = hundreds of nmol L^-1^ for individual polysulfides, Kamyshny et al. (2006)) in all samples. The analyses of thiosulfate and sulfite were carried out simultaneously by the same HPLC analysis procedure as for low hydrogen sulfide concentrations. The MDL was 2 nmol L^-1^. Free thiocyanate (SCN^-^) and tetrathionate (S_4_O_6_^2-^) concentrations were below the detection limit (60 nmol L^-1^ and 500 nmol L^-1^, respectively) of chromatographic analyses (Rong et al., 2005; Kamyshny, 2009a). Sulfate (SO_4_^2-^) was quantified by ion chromatography (DIONEX, DX500) with ASRS-300 suppressor. The eluent, which was composed of 1.8 mmol L^-1^ sodium carbonate and 1.7 mmol L^-1^ sodium bicarbonate, was pumped at a flow rate of 2 mL min^-1^ through a guard column (AG4A-SC), an anion exchange column (AS4A-SC). The MDL of this method was 10 μmol L^-1^.

### Solid Phase Analysis

Sediments were sliced in the field at 2 cm intervals using a core extruder and a spatula. For acid-volatile sulfur (hereafter AVS), non-S^0^ chromium reducible sulfur (CRS) and elemental sulfur measurements, c.a. 15 mL of sediment were placed in a 50 mL volume falcon tube containing 25 mL zinc acetate in the field (50 g L^-1^). Additionally, for metal speciation measurements, c.a. 25 mL of sediments were placed in a 50 mL falcon tube and frozen the same day on arrival to the laboratory. For porosity measurements, 10 mL of sediment were placed in a 15 mL volume falcon tube and stored at 4°C in the laboratory.

Elemental sulfur was extracted from the sediment that had been pre-treated with zinc acetate by shaking with methanol for 16 h on a rotary shaker (Zopfi et al., 2004). The sample-to-methanol ratio was c.a. 1/20 [w/v]. After separation of the sediment by centrifugation, elemental sulfur (S^0^) in the methanol extract was quantified by HPLC. An Agilent Quaternary pump – G1311B, UV-VIS Detector (Agilent G1365D), and Prevail C18 Grace reverse phase column (250 mm × 4.6 mm × 5 μm) were used. Pure methanol (HPLC grade) was used as a mobile phase at a flow rate of 1 mL per minute. Elemental sulfur was detected at 230 nm wavelength with an MDL of 10 μmol kg^-1^ (wet sediment). In two samples of surface sediments at site RS-V-21, the elemental sulfur content was high enough to allow analysis of isotopic composition. Elemental sulfur was preconcentrated by partial evaporation of methanol on a rotary evaporator, extraction with dichloromethane from the residual methanol-water mixture and evaporation of dichloromethane under gentle flow of nitrogen. Elemental sulfur was reduced to hydrogen sulfide by Cr(II) reduction in the ethanol-water medium according to Gröger et al. (2009). Evolving hydrogen sulfide was trapped in AgNO_3_/HNO_3_ trapping solution.

Acid-volatile sulfur and CRS distillations were performed on the sediment sample after it had been subjected to methanol extraction, so that elemental sulfur was not accounted for in the CRS distillation. Although greigite (Fe_3_S_4_) is an important precursor for pyrite, its content in sediments is usually low (Morse and Cornwell, 1987). Thus, the main sulfur mineral recovered by CRS was pyrite sulfur, which will be referred to as pyrite-S (or Py-S) hereafter. The sediments were subjected to a two-step distillation. AVS was extracted over two hours by boiling in 5 M HCl, which was followed by 3 h boiling with 1 mol L^-1^ acidic CrCl_2_ solution (extracting CRS) (Fossing and Jørgensen, 1989). The MDL was 10 μmol kg^-1^ wet sediment or 50 nmol total pyrite-S.

Total iron and manganese concentrations were measured according to Aller et al. (1986). For quantification of iron and manganese SpectrAA 300/400 Series Varian AAS was used. Results are presented in moles of metal per kilogram of wet sediment after correction based on sediment porosity. The MDL was 2 μmol kg^-1^ (wet sediment) for iron and 0.6 μmol kg^-1^ (wet sediment) for manganese. For quantification of reactive iron species (Fe_HR_) a sequential extraction scheme was used according to Poulton and Canfield (2005). Quantification was performed by AAS, and the MDL was 2 μmol kg^-1^ Fe (wet sediment). Pyritic iron was measured on a subsample by the CRS protocol.

For porosity analysis, the weight and volume of wet sediment and of sediment dried at 50°C for one week were measured. The porosity was calculated as the fraction of pore-water volume in the wet sediment. Total organic carbon (TOC) was quantified by an elemental analyzer (LECO SC632). The MDL was 0.005% TOC with precision of 1%.

### Isotopic Analysis

For preparation for sulfur isotopic analysis of AVS and pyrite-S, zinc sulfide that was formed during hydrogen sulfide distillation was converted to silver sulfide by addition of an excess of silver nitrate solution. Each Ag_2_S sample was aged for at least one week and cleaned with sequential washes of 4 × 50 mL Milli-Q water, rinsed in 50 mL of 1 M NH_4_OH overnight, then washed with 3 × 50 mL Milli-Q water and dried at 55°C. For the isotopic analysis of sulfate, sediments were centrifuged, the supernatant was filtered, and an excess of barium chloride solution was added to the filtrate in order to precipitate BaSO_4_. The precipitate was washed with 3 × 50 mL Milli-Q water and dried at 55°C.

For the analysis of δ^18^O_SO4_, barium sulfate was pyrolyzed at 1450°C in a temperature conversion element analyzer (TC/EA), producing carbon monoxide. Carbon monoxide was measured by continuous helium flow in a GS-IRMS (Thermo Finnegan Delta V Plus, at the Godwin Laboratory, University of Cambridge). Analyses of δ^18^O_SO4_ were performed in replicates (n = 3–5) and the standard deviation of the replicate analysis is reported (∼0.4‰ 1σ). To analyze the δ^34^S_SO4_, δ^34^S_Py-S_, δ^34^S_AV S_, δ^34^S_S0_ values, barite or silver sulfide with an excess of vanadium pentoxide were combusted at 1030°C in a flash element analyzer (EA), and the resulting sulfur dioxide (SO_2_) was measured by continuous helium flow on a GS-IRMS (Thermo Finnegan Delta V Plus Godwin Laboratory, University of Cambridge). The analytical error for the δ^34^S analysis was determined using the standard deviation of standards run at the beginning and the end of each run (long term reproducibility of our standards is ∼0.3‰ 1σ). δ^18^O_SO4_ is reported relative to the Vienna Standard Mean Ocean Water (VSMOW) and δ^34^S is reported with respect to Vienna Canyon Diablo Troilite (VCDT).

## Results

### Sediment Description

In the vicinity of the north shore of the Gulf of Aqaba (sites RS-II-19 and RS-V-21), sediments contain grayish sandy particles, suggesting the source of the sediment is terrigenous material derived from adjacent rocks by erosion and/or weathering and transported by seasonal floods. The sediments from near the fish farm contained black fragments which are likely residual material from fish cages, consistent with the composition of sediment cores collected during the operation period of Ardag fish farm (Katz et al., 2002). The bulk composition of the north shore sediments is dominated by siliciclastic material with lower carbonate content (6.17%; Al-Rousan et al., 2006) compared to coral reef sediments (74.8%; Al-Rousan, 1998). The sediment from the intermediate and deep-water sites (RS-IV-306, RS-III-420, and RS-I-694) is characterized by a much finer grain size with a higher content of light brown clay and mud. These textural properties at the deeper-water sites reflect the aeolian source of particles derived from desert soils (Garrison et al., 2003).

### Pore-Water Chemistry

**FIGURE 2:**
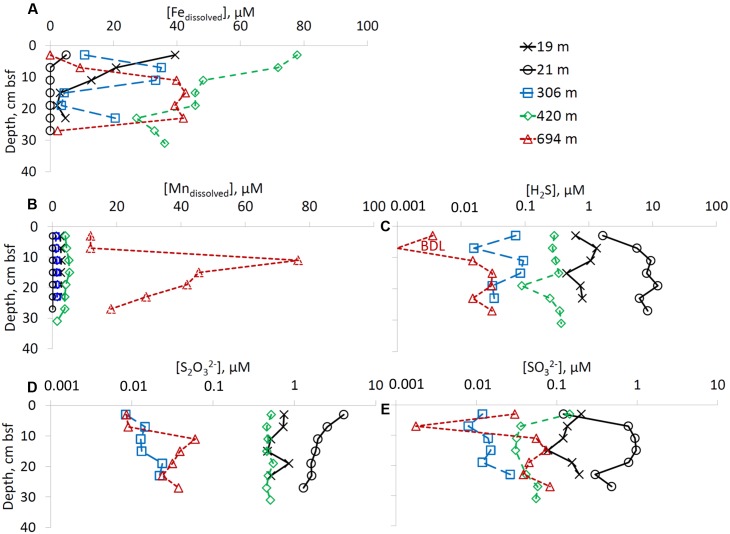
Pore-water chemistry. Profiles of **(A)** dissolved iron, **(B)** dissolved manganese, **(C)** hydrogen sulfide (pay attention to logarithmic x-axis), **(D)** thiosulfate (pay attention to logarithmic x-axis), and **(E)** sulfite (pay attention to logarithmic x-axis). Right upper panel presnets a color codes for sampling sites.

Dissolved iron was present in the sediments of the deep-water sites (RS-IV-306, RS-III-420, and RS-I-694) at all depths below seafloor, while at the shallow water site that were not influenced by the fish farms (RS-V-21) no dissolved iron was detected below 3 cm below seafloor (**Figure 2A**). Dissolved manganese concentrations increase with overlying water column depth. Concentrations above 10 μmol L^-1^ were detectd only at the deepest site RS-I-694 (**Figure 2B**).

Sulfate concentrations were constant with depth at all the sampling sites, in the range of 32–35 mmol L^-1^ (data not shown). Hydrogen sulfide concentrations were below 0.34 μmol L^-1^ at the deep-water sites (RS-IV-306, RS-III-420 and RS-I-694), up to 1.3 μmol L^-1^ at former fish farm site RS-II-19, and up to 12 μmol L^-1^ at site RS-V-21 (**Figure 2C**). Zero valent sulfur (ZVS) was below detection limit at sites RS-II-19 and RS-IV-306, below 200 nmol L^-1^ at sites RS-III-420 and RS-I-694 and up to 301 nmol L^-1^ at site RS-V-21 (data not shown). The highest thiosulfate concentrations were detected at the shallow water sites (up to 4.05 μmol L^-1^ at site RS-V-21). At sites RS-IV-306 and RS-I-694, thiosulfate concentrations were <0.1 μmol L^-1^ (**Figure 2D**). Sulfite concentrations in the pore-waters were lower than thiosulfate concentrations. At the shallow sites (RS-II-19 and RS-V-21), sulfite concentrations were 78–972 nmol L^-1^. Sulfite concentrations at the deeper sites were <150 nmol L^-1^ (**Figure 2E**).

### Solid Phase Chemistry

**FIGURE 3:**
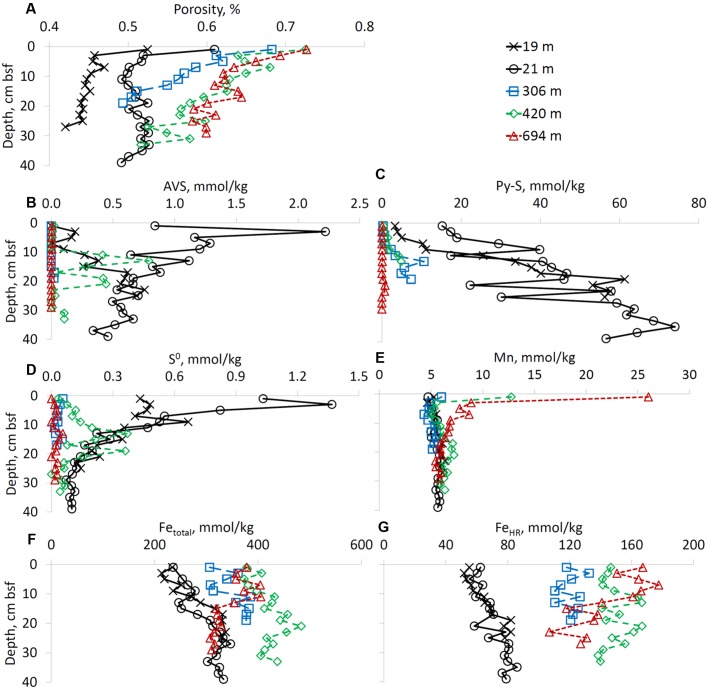
Sediment porosity and solid-phase composition (except iron speciation). Profiles of **(A)** porosity, **(B)** AVS, **(C)** pyrite sulfur, **(D)** elemental sulfur, **(E)** manganese, **(F)** total iron, and **(G)** sum of “highly reactive” iron species. Right upper panel presnets a color codes for sampling sites. All concentrations are given in mmol kg^-1^ of wet sediment.

The porosity of the sediments increased with increasing water depth as well as with depth below the sediment-water interface (**Figure 3A**). TOC content was in the range of 0.1-0.6 wt% at all sites. The AVS and pyrites sulfur contents of the sediment decreased with an increase in water column depth. At the shallow site not influenced by anthropogenic pollution (RS-V-21), AVS content decreased with depth below the seafloor and pyrite sulfur increased with depth below the seafloor. At site RS-II-19 (the site impacted by the fish farm) the lowest AVS concentration was detected in the upper 10 cm of sediments (**Figures 3B,C**). In the upper 10 cm of the sediment, elemental sulfur content was higher at the shallow water sites than at the deep-water sites (**Figure 3D**).

Sediments from the deep-water sites were enriched in total manganese in the upper two centimeters of the core. At RS-I-694 site total manganese content reached 26.0 mmol kg^-1^ of wet sediment. In cores taken from shallow-water sites (RS-II-19 and RS-V-21), the total manganese content was below 7 mmol kg^-1^ of wet sediment (**Figure 3E**). Sediments in the deep-water sites were enriched in iron compared to shallow-water sites, especially in the upper 14 cm of the sediment. The highest total iron content was detected at site RS-II-420 (**Figure 3F**). Traditionally, highly reactive iron is defined as the sum of iron species which are reactive toward hydrogen sulfide and iron which has already reacted with sulfide (e.g., pyrite iron). The content of highly reactive iron, which in our study includes pyrite iron, acetate-, hydroxylamine-, dithionite-, and oxalate-extractable iron, increases with water depth (**Figure 3G**). As concentrations of pyrite are higher at the shallow-water sites (**Figure 3C**), concentrations of iron species which are still reactive toward hydrogen sulfide increase further with water depth. At the shallow-water sites (RS-II-19 and RS-V-21) concentrations of highly reactive iron slightly increased with depth (**Figure 3G**). Sodium acetate-extractable iron, which accounts for Fe(II) carbonate minerals such as siderite and ankerite represented less than 10% of the highly reactive iron at all water depths. Dithionite-extractable iron (goethite + hematite + akaganéite) represented the most abundant fraction of highly reactive iron in the sediments of the Gulf of Aqaba (data not shown).

### Isotopic Composition of Sulfur Species

**FIGURE 4:**
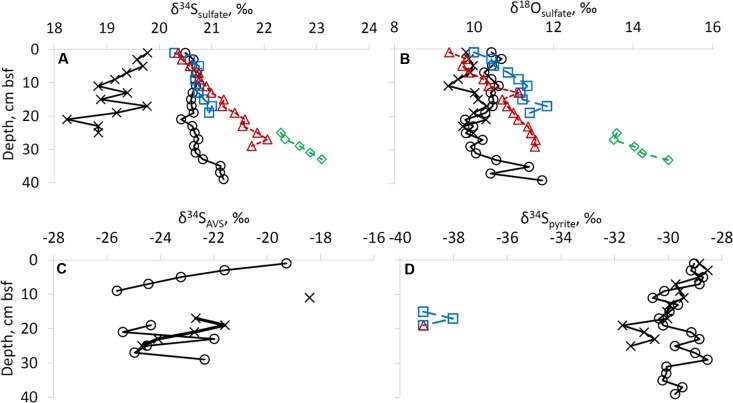
Isotopic composition of sulfur species. Profiles of **(A)** δ^34^S of sulfate, **(B)** δ^18^O of sulfate, **(C)** δ^34^S of AVS, **(D)** δ^34^S of pyrite. For legend see right upper panel of **Figure 2**.

The isotopic composition of the Gulf of Aqaba seawater sulfate was measured in five samples. Three samples were taken at the surface at various locations and two samples were retrieved from 20 and 700 m depths. The isotope composition of sulfate at various locations and water depths within the Gulf of Aqaba is similar (δ^34^S = 20.3 ± 0.2‰, δ^18^O = 8.6 ± 0.2‰), and does not differ more than the error of the analytical method. At all sites, except at the former fish farm (site RS-II-19), the δ^34^S of sulfate near the top of the sediment was similar to the overlying water (20.3–20.5‰). At these sites, the δ^34^S increased with depth below the seafloor to 21.2–23.1‰. At the former fish farm site, sulfate sulfur was isotopically lower than seawater sulfate, possibly due to re-oxidation of isotopically light reduced sulfur minerals formed during fish farm operation (**Figure 4A**). At all depths bsf, the δ^18^O of sulfate was higher than seawater sulfate (9.4–10.4‰ in the upper 2 cm of sediment vs. 8.6% in the water column). For the deep-water sites, a slight increase in δ^18^O of sulfate was observed with depth (**Figure 4B**).

The sulfur isotopic composition of AVS is only reported for the shallow-water sites, as at the deep-water sites the AVS content was too low to perform sulfur isotopic analysis. The most ^34^S-enriched AVS was detected at both shallow-water sites in the uppermost samples: δ^34^S = –18.4‰ to –19.3‰. At 17–29 cm bsf, the δ^34^S of AVS was in the range of –21.6‰ to –25.4‰ (**Figure 4C**). Enough pyrite-S for sufur isotopic analysis was retrieved from all sediments at the two shallow-water sites, three samples from site RS-IV-306 from 14 to 20 cm, and one sample from site RS-I-694 at 18–20 cm (this point cannot be seen on the figure as it coincides with the point for RS-IV-306 profile). Pyrite sulfur was isotopically lower in δ^34^S than AVS sulfur. At deepwater sites RS-IV-306 and RS-I-694, pyrite was isotopically lower in δ^34^S than at the shallow-water sites (**Figure 4D**). The isotopic composition of elemental sulfur was measured in the 0–2 cm and 2–4 cm intervals in the sediments from site RS-V-21and the δ^34^S was –20.9‰ and –22.4‰, respectively.

## Discussion

### Manganese and Iron Cycles

The concentration of total manganese in the solid sediment increases with the depth of the overlying water column (**Figure 3E**). This increase is likely caused by greater contribution of aeolian dust to the sediment further from the shore. The atmospherically transported material from the Sahara and adjacent deserts has a high manganese content, as much as 880 ppm (16 mmol kg^-1^) in Saharan dust (Mendez et al., 2010). In contrast, the shallow sediment has a dominance of fluvial input with lower manganese content. An enrichment in solid manganese in the upper 2 cm of the sediment was observed in the deep-water sites (**Figure 3E**). This solid manganese enrichment results from the oxidation of reduced aqueous manganese (Mn^2+^), which is produced by microbial manganese reduction coupled to organic carbon oxidation. This reduced Mn^2+^ produced deeper in the sediment diffuses toward the water-sediment interface where it is oxidized to manganese oxides. The only site at which concentrations of dissolved manganese were higher than concentrations of dissolved iron was deepwater site RS-I-694 suggesting a dominance of the manganese cycle at this location.

The concentration of total sedimentary solid iron, similar to total manganese, increases as the depth of the overlying water column increases, except for the deepest site (RS-I-694) (**Figure 3F**). As with manganese, this increase may be explained by greater contribution of aeolian dust away from the shore. The fine-grain sediment that is transported from the Sahara and adjacent deserts by wind has a high iron content (Chase et al., 2006). The lack of a significant increase of solid total iron in the surface sediment layer is likely due to low iron sulfide solubility, which causes retention of iron in the sediment even at low concentrations of hydrogen sulfide in the pore-waters (**Figures 2A,C**).

Highly reactive iron is traditionally defined as the fraction of iron in the sediment that can react with hydrogen sulfide to produce FeS or FeS_2_ (Berner, 1970). There are different pools of iron that are defined based on their reactivity toward hydrogen sulfide. Ferrihydrite and lepidocrocite react with hydrogen sulfide within hours. The most abundant iron minerals in dust from the Saharan Desert, such as hematite and goethite, react with hydrogen sulfide on time scales of days (Poulton et al., 2004) and years (magnetite) (Canfield et al., 1992). For iron in sheet-silicate minerals it may take up to millions of years to react with hydrogen sulfide (Canfield et al., 1992; Raiswell and Canfield, 1996). Highly reactive iron concentrations are lower at the shallow water sites (RS-II-19 and RS-V-21) than at the sites overlain by deeper waters. We suggest that the difference in the reactive iron content could be due to the difference in the highly reactive iron content of dust (relatively high) and desert soil and rock material transported by flash floods (relatively low), which predominantly impact shallow-water sediments. Although detailed studies of reactive iron speciation of minerals and soils in the various locations in arid Arava valley are not yet published, total iron content in granite was recently reported for two locations near Eilat: Wadi Shelomo and Mt. Rehavam with 0.30–0.76% Fe_2_O_3_ and 0.12–1.60% Fe_2_O_3_, respectively (Eyal et al., 2010). The iron in these locations is mostly associated with biotite (Bogoch et al., 1997). The detailed study of iron speciation in the seasonal stream beds, which is out of scope of this study, should be performed in order to evaluate this hypothesis.

### Sulfur Cycle

The concentration of hydrogen sulfide in the pore-water decreased with an increase in water column depth from 0.5 to 12 μmol L^-1^ at the shallow-water sites to ≤30 nmol L^-1^ at site RS-I-694. The latter concentration was detected by HPLC with a fluorescence detector and is a concentration we usually consider to be below the detection limit of standard spectrophotometric techniques (1 μmol L^-1^ – Cline, 1969). Despite extremely low concentrations of hydrogen sulfide in the deeper sediments, we use three lines of evidence to confirm the presence of a sedimentary sulfur cycle in the Gulf of Aqaba.

#### Pyrite Content

The first line of evidence for microbial sulfate reduction is the presence of pyrite. Pyrite is formed by two mechanisms, both involving hydrogen sulfide: the “polysulfide” mechanism (Eqs. 1–3) (Luther, 1991; Kamyshny et al., 2004) and the “sulfide” mechanism (Eqs. 1, 4) (Rickard and Luther, 1997; Gartman and Luther, 2013).

$$2\text{FeOOH+3}H_{2}S\to 2FeS+S^{0}+4H_{2}O$$

$$H_{2}S+(n-1)S^{0}\overset{\leftarrow }{\to }2H^{+}+S_{n}^{2-}$$

$$FeS+S_{n}^{2-}\to FeS_{2}+S_{n-1}^{2-}$$

$$FeS+H_{2}S\to FeS_{2}+H_{2}\,$$

In our case, circumneutral pH and the presence of elemental sulfur in the sediment (**Figure 3D**), suggest that the “polysulfide” mechanism is the most feasible pathway of pyrite formation. Although concentrations of polysulfides in the pore-waters were below the detection limit of our analytical technique (Kamyshny et al., 2006), it is likely that their concentration was low due to their fast reaction during pyrite precipitation. Oxidation of polysulfides by oxygen is known to be faster than oxidation of hydrogen sulfide by oxygen (Kleinjan et al., 2005). Thus, concentrations of polysulfides in various natural aquatic systems are better explained by kinetics of their reaction rather than by thermodynamic considerations (Kamyshny and Ferdelman, 2010; Lichtschlag et al., 2013).

Sedimentary pyrite content decreases with increasing water column depth and with depth below the sediment-water interface (**Figure 3C**). Depth-based net pyrite formation rates were calculated from the linear approximation of the increase of pyrite concentrations with depth, and converted to time-based rates using the typical sedimentation rate for deep sites (0.054 cm year^-1^ – Al-Rousan et al., 2004). Net pyrite formation rates were estimated to be 0.68, 0.53, 0.11, 0.073, and 0.0063 nmol cm^-3^ wet sediment day^-1^ at sites RS-II-19, RS-V-21, RS-IV-306, RS-III-420, and RS-I-694, respectively. These rates of pyrite formation are lower than rates of microbial sulfate reduction in most marine sediments. One explanation of this observation is re-oxidation of a significant portion of hydrogen sulfide formed during microbial sulfate reduction. Reoxidation of hydrogen sulfide to sulfide oxidation intermeditates, which in turn may be microbially disproportionated to sulfate and hydrogen sulfide allows only trace amounts of pyrite to form. On the other hand, the higher highly reactive iron content at the deeper sites (**Figure 3G**) may give preference to iron reducing microorganisms, thus suppressing microbial sulfate reduction.

#### Sulfide Oxidation Intermediates

The second line of evidence for an active sulfur redox cycle in the sediments of the Gulf of Aqaba is the presence of intermediate-valence state sulfur species. Hydrogen sulfide is a precursor to sulfide oxidation intermediates such as zero-valent sulfur, thiosulfate and sulfite in sedimentary pore-waters (**Figures 2D,E**) as well as of zero-valent sulfur in the solid phase (**Figure 3D**). Thus, the presence of intermediate sulfur species in the sedimentary pore fluids may be interpreted as an argument for chemical or microbial oxidation of hydrogen sulfide.

Zopfi et al. (2004) suggested that concentrations of sulfide oxidation intermediates in marine sediments are controlled by their availability for microbial metabolism. Usually, the concentration of these intermediates decreases in the order [S^0^] > [S_2_O_3_^2-^] > [SO_3_^2-^] > [S_4_O_6_^2-^], as we observe in the sediments of the Gulf of Aqaba (**Figures 2D,E**, **3D**). As expected, the concentration of elemental sulfur in the sediments decreases with increasing water column depth, e.g., with the decrease in the rates of formation of its precursor, hydrogen sulfide. In the shallow-water sites, the concentration of elemental sulfur decreases with sediment depth as well, due to the formation of pyrite which will consume the available hydrogen sulfide. At the shallow-water sites, the concentration of elemental sulfur reaches 1 mmol kg^-1^ of wet sediment. Only colloidal sulfur with particles <150 nm, polysulfide sulfur, and dissolved elemental sulfur can pass the membrane of the Rhizon samplers. Sulfur is predominantly in the solid sediment, as zero-valent sulfur concentrations in pore-waters are <300 nmol L^-1^ at all sites and sediment depths. As the solubility of elemental sulfur in seawater at 25°C is 147 nmol L^-1^ (Kamyshny, 2009b), at some sites the presense of polysulfides is required to explain the observed results in the porewater, as colloidal sulfur equilibrates with hydrogen sulfide according to Eq. (2) in minutes to hours (Fossing and Jørgensen, 1990; Kamyshny and Ferdelman, 2010).

The concentration of thiosulfate in the pore-waters decreases with increasing water depth as is expected due to the corresponding decrease of hydrogen sulfide concentrations. A similar trend was observed for concentrations of sulfite. The concentrations of thiosulfate and sulfite are typical for marine sediments, although, it must be mentioned that in marine sediments, hydrogen sulfide concentrations are often much higher than our results for the Gulf of Aqaba (Thamdrup et al., 1994; Zopfi et al., 2004, 2008; Lichtschlag et al., 2013).

**FIGURE 5:**
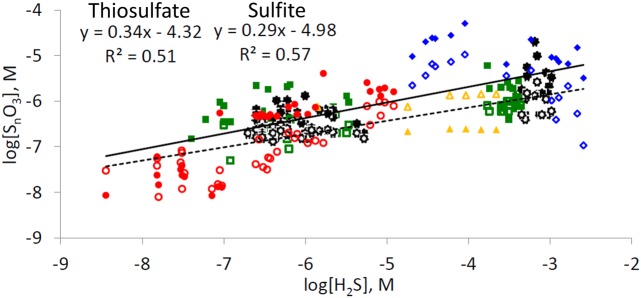
Dependence of concentrations of thiosulfate (closed symbols) and sulfite (opened symbols) on concentrations of hydrogen sulfide in marine sediments (not including salt marshes and tidal flats). Red circles – this work from the Gulf of Aqaba, orange triangles – results from Thamdrup et al. (1994) from Aarhus Bay sediments, green rectangles – results from Zopfi et al. (2004) from the Black Sea, black stars – results from Zopfi et al. (2008) from the upwelling area off central Chile coast, blue diamonds – results from Lichtschlag et al. (2013) from Dvurechenskii mud volcano, Black Sea. Solid line represents a trendline for all thiosulfate vs. hydrogen sulide data points, dashed line represents a trendline for all sulfite vs. hydrogen sulfide datapoints. Only data which were obtained by chromatographic analysis after 2,2′-dithiobis(5-nitropyridine) derivatization (Valravamurthy and Mopper, 1990) and monobromobimane derivatization (see Materials and Methods section and references therein) for points where both hydrogen sulfide and sulfur oxoanion concentrations were above the detection limit are presented.

The co-dependences of the concentration of thiosulfate and sulfite on the concentration of their precursor - hydrogen sulfide - produces a rather unexpected relationship (**Figure 5**). The increase in the concentration of thiosulfate and sulfite in pore-waters is often more moderate than the increase in hydrogen sulfide concentrations: a 10-fold increase in hydrogen sulfide leads to only c.a. fivefold increase in concentration of intermediate sulfur oxyanions. The simultaneous presence of hydrogen sulfide and intermediate oxyanions in marine sediments is possible only if two conditions are fullfilled: (1) low or zero dissolved oxygen levels are present in pore-waters; (2) if oxygen is absent, other electron acceptors, capable of chemical or microbial oxidation of hydrogen sulfide (e.g., nitrate, MnO_2_, Fe(III) (hydr)oxides)), are present in the sediment. As nitrate concentrations in seawater of the Northern Gulf of Aqaba are low (<6 μmol L^-1^) (Al-Qutob et al., 2002) and it is depleted quickly in the pore-waters, manganese and iron oxides are the main electron acceptors responsible for hydrogen sulfide oxidation in these locations. Chemical oxidation of hydrogen sulfide by Mn(IV) leads to formation of zero-valence sulfur, thiosulfate, sulfite, and sulfate (Burdige and Nealson, 1986), and chemical oxidation of hydrogen sulfide by Fe(III) forms mostly zero-valence sulfur, although the formation of other sulfide oxidation intermediates has been documented (Yao and Millero, 1996; Wan et al., 2014). Therefore, the distribution of sulfide oxidation intermediates depends on the ratio of hydrogen sulfide-to-electron acceptor, where at lower ratios more oxidized products are formed. These reactions are first order with respect to both hydrogen sulfide and the concentration of electron acceptor. Microbial disproportionation and oxidation of intermediate sulfur species may lead to the formation of other sulfide oxidation intermediates as well as of the terminal oxidation product, sulfate (Bak and Cypionka, 1987; Jørgensen, 1990a,b; Zopfi et al., 2004, and references therein).

We suggest the following explanation for the observed trends. The decrease in hydrogen sulfide concentration in pore-waters leads to a decrease in the ratio between hydrogen sulfide and the concentrations of reactive iron and manganese oxides in the sediment. This, in turn, leads to formation of more oxidized sulfide oxidation intermediates (e.g., more sulfite and thiosulfate and less zero-valent sulfur species). On the other hand, a decrease in the concentration of intermediate sulfur oxyanions, leads to a decrease in the rates of their chemical and microbial consumption. Thus, an increase in the fraction of hydrogen sulfide that is converted to sulfur oxyanions, combined with a decrease in their consumption rate, results in only a moderate decrease in their concentrations with a corresponding decrease in hydrogen sulfide concentrations. In the extreme case of the sediments of the Gulf of Aqaba, concentration ratios of up to 4 and 8 between thiosulfate and sulfite, respectively, to hydrogen sulfide were found at site RS-I-694.

#### Isotopic Composition of Sulfur Species

**FIGURE 6:**
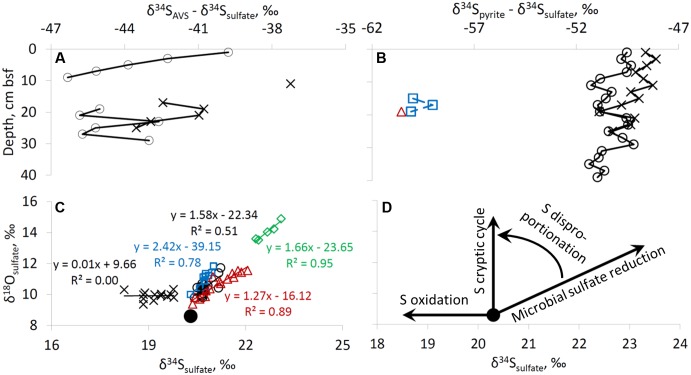
Difference between δ^34^S values of AVS and sulfate **(A)**, and of pyrite and sulfate **(B)**. Plot of δ^18^O of sulfate vs. δ^34^S of sulfate is presented in plate **(C)**. Schematic pathways of dependence of δ^34^S and δ^18^O values of sulfate on microbial sulfur transformation process is presented in the panel **(D)**. Large black circle in panels **(C,D)** represents isotopic composition of sulfate in the water column of the Red Sea. For legend see right upper panel of **Figure 2**.

The third line of evidence for microbial sulfur cycling in the sediments is the isotopic composition of sulfur species. In this study, the sulfur isotopic fractionation between sulfate and AVS (which represents the sulfur isotopic composition of the most recently formed hydrogen sulfide) is 37–46‰ (**Figure 6A**). Usually, in marine sediments both AVS and pyrite become isotopically more enriched in ^34^S with depth due to Rayleigh distillation of sulfur isotopes during microbial sulfate reduction and the diffusion of sulfate within pore-fluids. In our case, Rayleigh distillation cannot be invoked to explain the observed trends as (1) sulfate concentrations do not decrease significantly with depth, (2) AVS becomes lower in δ^34^S with sediment depth, and (3) pyrite sulfur becomes isotopically lower in δ^34^S with sediment depth and near the sediment surface it is isotopically lower in δ^34^S than AVS by approximately 10‰. An increase in sulfur isotope fractionation with depth may be explained by a decrease in microbial sulfate reduction rates (Chambers et al., 1975; Canfield, 2001; Sim et al., 2011b), which results from preferential mineralization of more bioavailable organic matter in the uppermost sediments. The difference of approximately 10‰ in the sulfate-pyrite sulfur isotope fractionation between shallow-water and deep-water sites (**Figure 6B**) supports an increase in sulfur isotope fractionation with a decrease in the rate of formation of hydrogen sulfide. A possible explanation for the relatively constant δ^34^S of pyrite in the surface sediments is the impact of bioturbation (**Figure 3C**). Rates of bioturbation in the shallow-water site near the fish farm (derived from chlorophyll profiles) were previously shown to be in the range of 0.013–0.069 cm^2^ d^-1^ (Black et al., 2012). The sulfur isotopic composition and concentrations in the upper 6 cm of sediments at shallow-water sites are thus possibly homogenized by bioturbation (**Figures 3C**, **4D**) which exponentially decreases with depth (Steiner et al., 2016).

In two analyzed samples (0–2 cm and 2–4 cm bsf at site RS-V-21) elemental sulfur was lower in δ^34^S by 0.8–1.6‰ than AVS. These results suggest that equilibrium in the H_2_S-polysulfide-S^0^ system is not achieved, as at the equilibrium zero-valent sulfur is higher in δ^34^S than hydrogen sulfide by up to 4‰ (Amrani et al., 2006; Kamyshny et al., 2014).

The relative change in the δ^34^S and δ^18^O of sulfate during microbial sulfate reduction has been shown to yield insight into the sulfur cycle in marine sediments and in laboratory cultures. At all the sites, except for RS-V-21, there is a coupled increase in δ^18^O and δ^34^S in sulfate as expected during microbial sulfate reduction. The relative change in δ^18^O vs. δ^34^S has been shown to correlate with the rate of microbial sulfate reduction (Böttcher et al., 1999; Aharon and Fu, 2003; Antler et al., 2013): the slower the sulfate reduction rate, the steeper the slope. The slopes in our sampling sites range between 1.2 ± 0.1 (at 694 m) to 2.4 ± 0.5 (at 306 m). It seems, however, that there is a discrepancy in the correlation between the slope and the sulfate reduction rates, as the most moderate slope is in the site where sulfate reduction rate is expected to be the lowest (694 m). We suggest that the disproportionation of intermediates of hydrogen sulfide oxidation increases the measured slope between δ^18^O and δ^34^S (Böttcher et al., 2005) (**Figures 6C,D**). At the former fish farm contaminated site (RS-II-19), a decrease in δ^34^S was observed together with a scattered change in the δ^18^O of sulfate (±0.6 ‰). This observation may be explained by more intense oxidation of reduced sulfur species at this site (**Figures 6C,D**). Combined with other evidence, the oxygen isotope composition of sulfate provides strong evidence for a presence of sulfur cycling in the sediments of the Gulf of Aqaba, including a near quantitative cycling of sulfur between oxidized and reduced or intermediate valence states with low net-consumption, which has been termed a ‘cryptic’ sulfur cycle.

### Impact of Aeolian Deposition on the Sulfur Cycle in the Sediments of the Gulf of Aqaba

**FIGURE 7:**
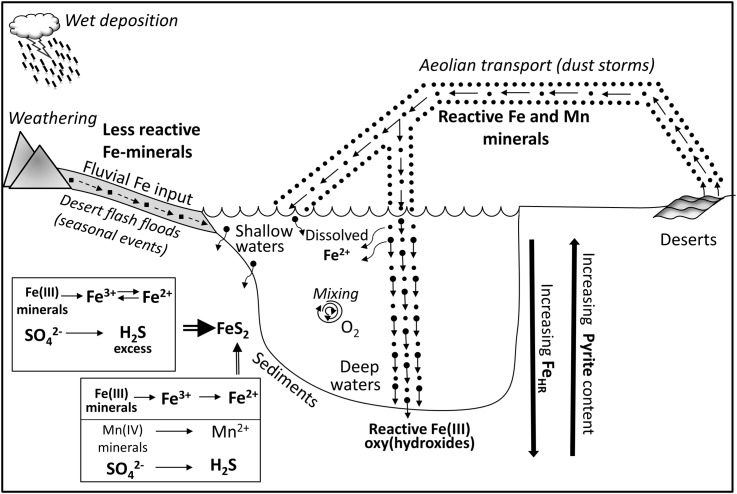
Schematic illustration of impacts of aeolian and fluvial sedimentary inputs on biogeochemistry redox-sensitive elements in the sediments of the Gulf of Aqaba. Aeolian input of highly reactive iron minerals is presented as a sum of fluxes of mineral dust which is derived from adjacent areas (rock weathering, secondary airflow) and dust which is transported from deserts by long distance aeolian transport (dust events, dust storms). Fluvial iron input is presented as sediment transport from adjacent areas (mainly Arava desert) by seasonal flashfloods which take place during winter rainfall events. The majority of wind-blown iron particles rapidly settle throughout the water column to the sediment, while minor fraction of reactive iron (III) oxyhydroxides dissolves in the water column. See text for detailed explanation of biogeochemical processes in the sediments.

An integrated scheme of processes affecting cycling of redox-sensitive elements in the Gulf of Aqaba is presented in **Figure 7**. Sediments overlain by deep water receive predominantly input of aeolian dust, which has high manganese and reactive iron content. At these water depths, microbial iron and manganese reduction is preferred over sulfate reduction. In spite of a microbial cycle dominated by iron and manganese cycling, there is microbial sulfate reduction and trace amounts of pyrite accumulating. However, the vast majority of the hydrogen sulfide produced is reoxidized in the presence of the high concentration of iron and manganese oxides to sulfide oxidation intermediates, which are further oxidized or disproportionated to form the terminal oxidation product, sulfate. These processes induce the high concentration of sulfide oxidation intermediates in pore waters and leave fingerprints in oxygen isotope composition of the sulfate. Thus, at these deep water sites the sulfur cycle is mostly cryptic.

Shallow water sites are influenced by a combination of aeolian dust input and fluvial iron and manganese inputs. At these sites, the manganese (especially close to the sediment-water interface) and reactive iron contents are lower than at the deep-water sites. Lower reactive iron content allows relatively high amounts of hyrdogen sulfide to be present in the pore-waters and to be further preserved in the form of pyrite.

In summary, this work shows that in arid environments, such as the Gulf of Aqaba, aeolian dry deposition from the surrounding deserts results in an increased flux of reactive iron and manganese to the sediments. Such increase leads to fast reoxidation of hydrogen sulfide and prevents pyrite formation in the sediments. Future work should concentrate on the detailed study of speciation of iron and manganese in the aeolian and fluvial sedimentary sources. Another direction of future research should include the study of impact of aeolian dry deposition on sedimentary sulfur cycle in other marine systems affected by aeolian dust deposition. Such research efforts are required in order to understand whether these considerations are unique to the Gulf of Aqaba or may be applied to other marine systems situated in arid regions.

## Author Contributions

BB and AK designed research; BB, VB, AT, GA, NK, RK and AK performed analyses; BB, VB, AT, GA, US, NK, RK and AK interpreted data, wrote the paper and approved the final version to be published.

## Conflict of Interest Statement

The authors declare that the research was conducted in the absence of any commercial or financial relationships that could be construed as a potential conflict of interest.

## Footnotes

1 http://www.geomapapp.org
